# Supplementary figures and images for: The shifting landscape of vaccine discourse: Insights from a decade of pre- to post-COVID-19 vaccine posts on social media
Source: PLoS One. 2025 Dec 19;20(12):e0337911. doi: 10.1371/journal.pone.0337911 (PMC12716706; doi:10.1371/journal.pone.0337911)

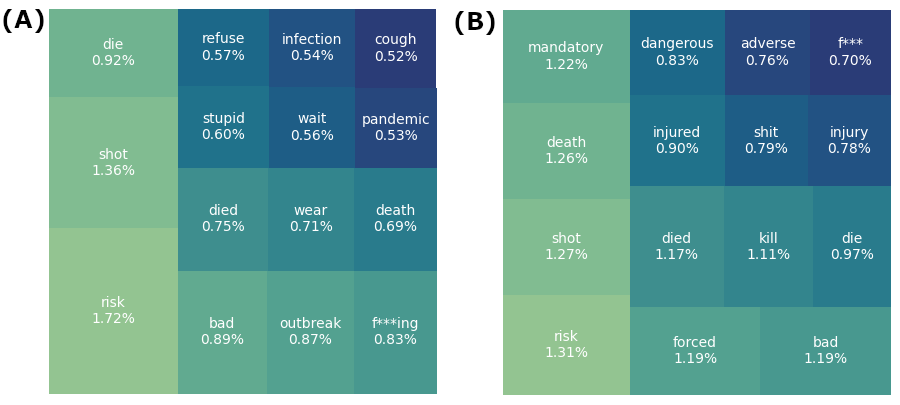

Supplement: S1 Fig — Treemap visualizations of the fifteen low-warmth words in the vaccine-related posts for (A) in-favor stance and (B) against stance. (TIFF) [file pone.0337911.s001.tif]

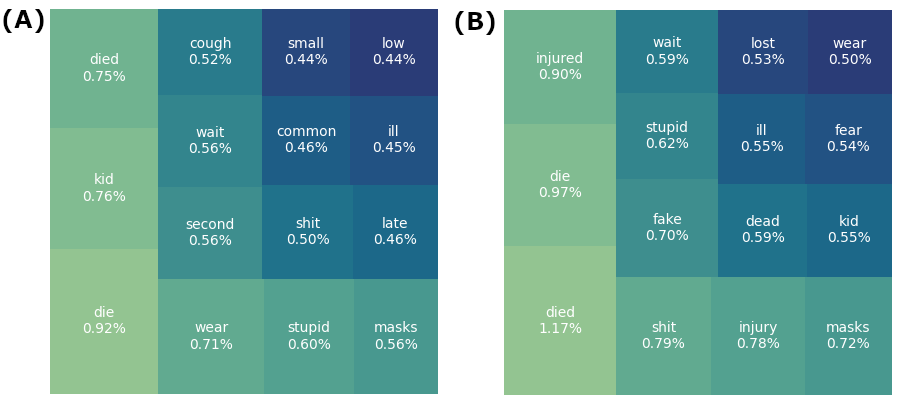

Supplement: S2 Fig — Treemap visualizations of the fifteen low-competence words in the vaccine-related posts for (A) in-favor stance and (B) against stance. (TIFF) [file pone.0337911.s002.tif]
