# Supplementary material for: The shifting landscape of vaccine discourse: Insights from a decade of pre- to post-COVID-19 vaccine posts on social media
Source: PLoS One. 2025 Dec 19;20(12):e0337911. doi: 10.1371/journal.pone.0337911 (PMC12716706; doi:10.1371/journal.pone.0337911)
Supplement: S1 Table — The related works are grouped based on their main topics–“Sentiment Analysis”, “Emotion Analysis”, “Topic Modeling”, “Stance Detection”, and “Vaccine misinformation and opinion mining”. (PDF) [file pone.0337911.s003.pdf]

S1 Table. Summary of related works on vaccines and COVID-19 post datasets

	Dataset	Total posts	Timeline
**Sentiment Analysis**			
Hu *et al.* [17]	COVID-19 English posts	308,755	Mar 1 2020–Feb 28 2021
Praveen, Ittamalla & Deepak [21]	COVID-19 posts	73,760	Sep 1–Dec 31 2020
De Rosis *et al.* [22]	COVID-19 Italian posts	774,407	Feb 17–Mar 22 2020
Yousefinaghani *et al.* [20]	COVID-19 posts	4,552,652	Jan 1 2020–Jan 31 2021
Stella, Restocchi & De Deyne [23]	COVID-19 Italian posts	101,767	Mar 11–Mar 17 2020
Alhuzali, Zhang & Ananiadou [24]	COVID-19 UK posts	516,427	Feb 1 2020–Nov 30 2021
Qorib *et al.* [25]	COVID-19 posts	42,796	Sep 26 2021–Nov 7 2021
Sarirete [26]	COVID-19 posts	230,623	Dec 16 2020–Jan 26 2021
Saleh *et al.* [27]	COVID-19 posts	2,287,344	Feb 1–Dec 11 2020
Burwell, Agarwal & Romine [30]	COVID-19 posts	916,686	Dec 1 2020–Feb 28 2021
**Emotion Analysis**			
De Rosis *et al.* [22]	COVID-19 Italian posts	774,407	Feb 17–Mar 22 2020
Stella, Restocchi & De Deyne [23]	COVID-19 Italian posts	101,767	Mar 11–Mar 17 2020
Alhuzali, Zhang & Ananiadou [24]	COVID-19 UK posts	516,427	Feb 1 2020–Nov 30 2021
**Topic Modelling**			
Hu *et al.* [17]	COVID-19 English posts	308,755	Mar 1 2020–Feb 28 2021
Praveen, Ittamalla & Deepak [21]	COVID-19 posts	73,760	Sep 1–Dec 31 2020
Saleh *et al.* [27]	COVID-19 posts	2,287,344	Feb 1–Dec 11 2020
**Stance Detection**			
D'Andrea *et al.* [18]	Italian vaccine posts	112,397	Sep 1 2016–Jun 30 2019
Giovanni *et al.* [19]	FR/DE/IT COVID-19 posts	70,352,366	Nov 1 2020–Nov 15 2021
Lindelöf, Aledavood & Keller [28]	COVID-19 posts	16,713,238	Mar 1 2020–Jul 31 2021
Mu *et al.* [29]	Labeled COVID-19 English posts	3,101	Oct 2020–May 2022
Alahmadi *et al.* [32]	Simulated anti-vaccine opinion network	5,000 agents (simulated)	Synthetic (simulation-based)
