# Supplementary material for: The shifting landscape of vaccine discourse: Insights from a decade of pre- to post-COVID-19 vaccine posts on social media
Source: PLoS One. 2025 Dec 19;20(12):e0337911. doi: 10.1371/journal.pone.0337911 (PMC12716706; doi:10.1371/journal.pone.0337911)
Supplement: S1 File — The prompt with “system” and “user” message was sent to the Llama 3.3 model to get stance classification. (DOCX) [file pone.0337911.s004.docx]

**Prompt for Llama 3.1 model**

To generate the stance of the author towards vaccines in a post, we used the following approach with the LLaMA model. The process involved sending a series of messages to the model, which included:

messages = [

{"role": "system", "content": "You are a very good stance detection model that can accurately detect the author's stance towards vaccines in a post."},

{"role": "user", "content": "What is the stance of the author of the following post towards vaccines? Reply 'favor' if the author is in favor of vaccines. Reply 'against' if the author is against vaccines. Reply 'neutral' if the stance cannot be determined. Remember to answer 'favor', 'against', or 'neutral' only. post=" + post}

]

Where, in this prompt:

- The system message instructs the model to act as a stance detection model with a focus on accurately identifying the author's stance towards vaccines.
- The user message contains the specific query for stance detection. It requests a classification of the post into one of three categories: favor, against, or neutral. The model is prompted to provide one of these responses based on the content of the post.
