# Supplementary material for: The shifting landscape of vaccine discourse: Insights from a decade of pre- to post-COVID-19 vaccine posts on social media
Source: PLoS One. 2025 Dec 19;20(12):e0337911. doi: 10.1371/journal.pone.0337911 (PMC12716706; doi:10.1371/journal.pone.0337911)
Supplement: S2 File — The file contains the vaccine timeline from COVID-19 virus discovery to vaccine development and boosters. Source: Wikipedia. (DOCX) [file pone.0337911.s005.docx]

**COVID-19 Vaccine Timeline^[1]^**

- **January 2020:** The COVID-19 pandemic starts with the identification of a new coronavirus in Wuhan, China.
- **March 2020:** Researchers begin working on developing vaccines for the virus.
- **April 2020:** Clinical trials for several vaccine candidates begin.
- **December 2020:** Emergency use authorization is granted for the first COVID-19 vaccine, developed by Pfizer and BioNTech.
- **January 2021:** More vaccines are granted emergency use authorization, including those developed by Moderna and AstraZeneca.
- **February 2021:** COVID-19 vaccination campaigns begin in many countries.
- **March 2021:** The World Health Organization (WHO) begins to roll out its COVAX initiative, which aims to provide equitable access to COVID-19 vaccines for all countries.
- **April 2021:** The WHO announces that the COVID-19 pandemic is a "once-in-a-century health crisis."
- **May 2021:** The WHO reports that over half of the world's population has received at least one dose of a COVID-19 vaccine.
- **June 2021:** More than 20 COVID-19 vaccine candidates are in clinical trials, and many countries have achieved high levels of vaccination coverage.
- **September 2021:** Some studies suggest that booster shots may be necessary to maintain protection against COVID-19, particularly for certain vaccines and in certain populations.
- **October 2021:** Some manufacturers, such as Pfizer and Moderna, begin clinical trials of booster shots for their respective vaccines.
- **November 2021:** The WHO announces that booster shots may be necessary for certain vaccines, and recommends that countries plan for booster campaigns as needed.
- **December 2021:** Some countries begin to roll out booster shot campaigns for certain populations, such as those who received the AstraZeneca vaccine earlier in the year.
- **January 2022:** Booster shot campaigns become more widespread, with many countries providing booster shots to those who have received a COVID-19 vaccine earlier in the year.
- **February 2022:** The WHO announces that booster shots are effective at increasing immunity to COVID-19, and recommends that countries prioritize booster shots for those at the highest risk of severe illness from the virus.

References

1. Wikipedia contributors. History of COVID-19 vaccine development; [Online; 730 accessed 27-March-2024]. https://en.wikipedia.org/wiki/History_of_COVID-19_vaccine_development.
